# Effects of Aromatherapy on Stress and Anxiety Among Intensive Care Unit Nurses: A Systematic Review

**DOI:** 10.1111/nicc.70705

**Published:** 2026-09-24

**Authors:** Abdullah Avcı, Mükaddes Avcı

**Affiliations:** ^1^ Anesthesia Program, Vocational School of Health Services Toros University Mersin Turkey; ^2^ Infection Control Committee Mersin University Hospital Mersin Turkey

**Keywords:** anxiety, aromatherapy, intensive care unit, nurses, stress

## Abstract

**Background:**

Nurses working in intensive care units (ICU) are at high risk for stress and anxiety. Aromatherapy stands out as a non‐pharmacological and easily implementable complementary approach for alleviating these symptoms. To our knowledge, this is the first systematic review specifically examining the effects of aromatherapy on stress and anxiety among ICU nurses.

**Aims:**

This systematic review aimed to evaluate the effects of aromatherapy on stress and anxiety among ICU nurses and to assess the methodological quality of the evidence.

**Study Design:**

In this systematic review, a literature search was conducted in the Scopus, PubMed, Web of Science and Cochrane Library databases without any time restrictions. The review includes randomised controlled, experimental and quasi‐experimental studies evaluating the effects of aromatherapy interventions on stress and/or anxiety among ICU nurses. The selection of studies, data extraction and assessment of methodological quality were conducted independently by two researchers.

**Results:**

This systematic review included five studies involving a total of 254 participants. Two of these studies are randomised controlled trials, and three are quasi‐experimental studies. The studies included in the review demonstrate that aromatherapy is administered using various essential oils and application methods (inhalation, topical application, etc.). The findings suggest that aromatherapy interventions may reduce stress among ICU nurses, whereas evidence regarding anxiety reduction is mixed and less consistent across studies.

**Conclusion:**

Aromatherapy may have potential benefits for reducing stress among ICU nurses, whereas evidence for anxiety reduction remains limited and inconsistent. Further high‐quality studies are needed before firm conclusions or routine clinical implementation can be recommended.

**Relevance to Clinical Practice:**

Aromatherapy may be considered as an optional supportive intervention within staff well‐being programs, provided that safety, scent sensitivity, infection‐control procedures and individual preferences are taken into account. However, standardised protocols and further high‐quality evidence are required to support its routine clinical use.

## Impact Statements

- What is known about the topic
  ○ ICU nurses experience high levels of stress and anxiety due to demanding and high‐risk clinical environments.
  ○ Aromatherapy is a commonly used complementary intervention with potential benefits for stress and anxiety reduction in healthcare professionals.
- What this paper adds
  ○ To our knowledge, this is the first systematic review specifically focusing on ICU nurses, a high‐risk and underexplored population.
  ○ Evidence for stress reduction was more consistent, whereas evidence for anxiety reduction remained limited and inconsistent across the included studies.
  ○ This review highlights the need for standardised aromatherapy protocols and well‐designed randomised controlled trials in ICU settings.

## 1 Introduction

Intensive care units (ICU) represent one of the most demanding and high‐stakes environments in healthcare, where nurses are continuously exposed to critically ill patients, life‐threatening conditions and complex clinical decision‐making processes. Alongside these responsibilities, factors such as staffing shortages, heavy workloads, shift‐based work systems and the emotionally charged nature of critical care create a sustained burden on nurses' physical and psychological well‐being. Consequently, ICU nurses are particularly vulnerable to elevated levels of stress and anxiety [1, 2, 3].

Stress and anxiety among ICU nurses have been widely recognised as significant occupational health concerns across different healthcare systems [4, 5]. Evidence suggests that persistent exposure to these stressors may impair psychological well‐being, reduce cognitive performance and negatively affect clinical decision‐making [6]. Moreover, high levels of occupational stress have been consistently associated with decreased work performance and increased risk of burnout among nurses in both developed and developing countries [7, 8]. These findings highlight the urgent need for effective, feasible and sustainable strategies to mitigate stress and anxiety in this high‐risk population.

In this context, non‐pharmacological and easily applicable interventions have gained increasing attention. Aromatherapy, defined as the therapeutic use of essential oils derived from plants, has emerged as a promising complementary approach due to its low cost, ease of application and minimal side effects. Aromatherapy may reduce stress and anxiety through olfactory stimulation and modulation of the limbic system, with potential effects on neuroendocrine pathways such as the hypothalamic–pituitary–adrenal axis [9, 10, 11]. Previous studies conducted among nurses and healthcare professionals in diverse clinical settings have suggested that aromatherapy may reduce occupational stress, anxiety and psychological distress [12, 13, 14]. However, much of this evidence derives from heterogeneous healthcare populations and non‐ICU settings, limiting its direct applicability to intensive care nurses.

Reducing stress and anxiety among ICU nurses is not only crucial for improving their psychological well‐being and job satisfaction but also for enhancing patient safety and quality of care. High levels of stress have been associated with decreased attention, impaired concentration and increased risk of clinical errors, while anxiety may negatively affect decision‐making processes and clinical judgement. Previous studies have demonstrated that occupational stress is significantly associated with cognitive failure and adverse events, as well as an increased likelihood of near‐miss errors among nurses, highlighting its direct implications for patient safety [15, 16]. Although aromatherapy has been investigated in broader nursing and healthcare populations, evidence specific to ICU nurses remains limited. To the best of our knowledge, no prior systematic review has specifically examined ICU nurses, who are exposed to uniquely high levels of occupational stress and anxiety. This distinction is particularly important, as the extreme workload, frequent exposure to life‐threatening situations and complex decision‐making processes characteristic of ICU settings may limit the generalisability of findings from other nursing populations. Therefore, interventions that are effective in lower‐intensity settings may not yield comparable outcomes in critical care environments. Addressing this gap is essential to provide context‐specific evidence and inform the development of targeted, feasible interventions for ICU nurses. In the light of this, this systematic review aims to critically evaluate and synthesise the available evidence on the effects of aromatherapy on stress and anxiety among ICU nurses, assess the methodological quality of included studies and identify existing gaps to inform future research and clinical practice. Accordingly, the following research question was addressed:

Is aromatherapy effective in reducing stress and anxiety levels among intensive care unit nurses?

## 2 Methods

This systematic review was conducted in accordance with the PRISMA (Preferred Reporting Items for Systematic Reviews and Meta‐Analyses) guidelines to address the research questions in a structured and transparent manner [17]. To reinforce our commitment to methodological rigour and transparency, the detailed protocol, prepared following the PRISMA Protocols (PRISMA‐P) statement, has been registered with PROSPERO (registration number: CRD420261285437).

### 2.1 Search Strategy

A comprehensive and systematic literature search was conducted in Scopus, PubMed, Web of Science and the Cochrane Library from database inception to June 6, 2026. The search strategy was developed in consultation with the review objectives and based on the Population–Intervention components of the PICOS framework. A combination of free‐text terms and Boolean operators was used to identify studies investigating the effects of aromatherapy among intensive care unit nurses. The following search string was adapted as necessary to the search syntax of each database: ((“intensive care unit*” OR ICU OR “critical care”) AND (nurse* OR nursing)) AND (aromatherapy OR “essential oil*” OR lavender). The complete database‐specific search strategies, including the search syntax, Boolean operators, truncation, field tags where applicable, and database‐specific adaptations, are provided in File S1.

**FIGURE 1:**
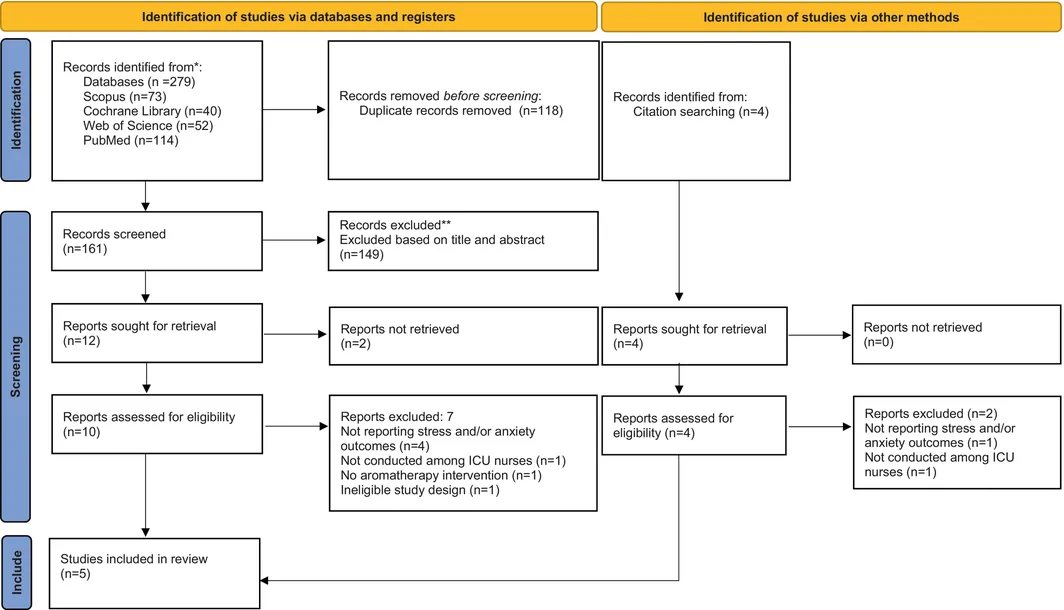
PRISMA 2020 flow diagram for new systematic reviews, which included searches of databases, registers and other sources.

Searches were conducted using the standard search interface of each database and adapted according to database‐specific search syntax. No restrictions were applied regarding publication date. To ensure feasibility and consistency in data extraction and interpretation, only studies published in English were considered eligible. To identify additional relevant studies and minimise the risk of missing eligible evidence, backward and forward citation searching of all included articles was performed manually. All searches were performed independently by two reviewers (AA and MA). Any discrepancies regarding the search process or study eligibility were resolved through discussion and consensus. The process of study selection, from a total of 279 records to the five studies included in the systematic review, is presented in a PRISMA flow diagram, with the number of studies categorised by database (Figure 1).

### 2.2 Study Selection

Study selection was conducted using the PICOS framework (Population, Intervention, Comparison, Outcome, Study design). Two independent reviewers (AA, MA) screened all titles and abstracts, followed by full‐text review of potentially eligible studies. Predefined inclusion and exclusion criteria were applied during study selection (Table 1). Any discrepancies between the reviewers were resolved through discussion and consensus.

**TABLE 1:** PICOS‐based inclusion and exclusion criteria.

Component	Inclusion criteria	Exclusion criteria
Population	ICU nurses; mixed samples included only when ICU data separately reported	Non‐ICU populations or mixed samples without separate ICU data.
Intervention	Aromatherapy interventions using therapeutic essential oils (inhalation, topical, gargle/mouthwash, etc.)	Non‐aromatherapy interventions or unclear aromatherapy protocols.
Comparison	Placebo, active comparator, usual care, no intervention or within‐subject comparison	Studies without a comparator and without pre–post outcome assessment
Outcome	Stress and/or anxiety measured using validated tools	Stress or anxiety not assessed, or non‐validated measures used.
Study design	RCTs, quasi‐experimental, non‐randomised, cross‐over, pre–post studies	Observational studies, qualitative studies, reviews, protocols, conference abstracts, case reports/series and other ineligible designs.

### 2.3 Data Extraction

First, the titles and abstracts of the studies were read and evaluated; subsequently, the articles for which the full text was available were examined in detail. The authors developed a standardised data extraction form for summarising the data, and the data were evaluated accordingly. Using a data extraction form, data were collected on the authors, year, location, study design, sample size, characteristics of the aromatherapy intervention, control condition, measurement tool and primary outcomes of the studies included in the systematic review. During the data extraction phase, the researchers independently entered the data into the data extraction form and then reviewed it together. In cases where researchers disagreed, a consensus was reached based on the existing literature.

### 2.4 Methodological Quality

The methodological quality of the included studies was assessed using the Joanna Briggs Institute (JBI) critical appraisal tools appropriate to each study design. The JBI Critical Appraisal Checklist for Randomized Controlled Trials (13 items) was used for randomised controlled trials, whereas the JBI Critical Appraisal Checklist for Quasi‐Experimental Studies (9 items) was applied to quasi‐experimental and non‐randomised studies [18, 19]. Each appraisal item was rated as ‘yes’, ‘no’, ‘unclear’ or ‘not applicable’. Quality appraisal was conducted independently by two reviewers, and any discrepancies were resolved through discussion and consensus. To ensure transparency, item‐level critical appraisal results for each study are presented in Tables 3 and 4. In addition, Table 2 provides a descriptive summary of the overall appraisal findings, including the number of JBI criteria met by each study. No studies were excluded based on methodological quality. Instead, the appraisal findings were considered when interpreting the overall strength and limitations of the evidence.

**TABLE 2:** Characteristics of included studies.

Author, year, country	Study design	Sample size	Intervention	Control condition	Outcome tools	Main findings	JBI appraisal result
Lee, 2023, South Korea	Randomised controlled trial (double‐blind, pretest–posttest)	AG: 29 CG: 28	Mode: Inhalation Essential oil: 3% *Origanum majorana* in almond oil Protocol: Inhaled during one ICU shift (2 h)	Almond oil placebo	VAS‐Stress, VAS‐Anxiety, STAI	Significant reduction in perceived stress and anxiety in the intervention group compared to baseline.	12/13
Eren, 2017, Turkey	Quasi‐experimental (self‐controlled, cross‐over design)	Cross‐over design (*n* = 45)	Mode: Inhalation Essential oil: Lavender Protocol: Five drops inhaled for 10–15 min during one day shift	Sunflower seed oil inhalation	PSS STAI‐II	No significant effect on stress or trait anxiety; state anxiety decreased in both intervention and control groups.	9/9
Pemberton, 2008, USA	Quasi‐experimental pilot study (pre–post, within‐subject sequential design)	Self‐controlled cross‐over (*n* = 14)	Mode: Topical Essential oil: *Lavandula angustifolia* 3% + Salvia sclaria 2% (5% total) in sweet almond oil Protocol: Applied once per shift over three 12‐h shifts	Sweet almond oil only; same application method and timing	Perceived stress (1–10 numeric rating scale)	Decrease in perceived stress levels during intervention shifts compared with control shifts	6/9
Seo, 2017, South Korea	Non‐randomised controlled clinical trial	AG: 40 PG:40 CG:40	Mode: Gargling Essential oil: Peppermint, lemon, tea tree, ylang (0.15%) Protocol: Once daily for 3 days	Saline gargling (placebo); No‐treatment control group	Visual Analog Scale (VAS, 0–10 cm)	Significant reduction in perceived stress in the aroma group compared with both saline and no‐treatment groups.	9/9
Hubbard, 2024, USA	Randomised, blinded, placebo‐controlled trial	AG: 10 PG: 8	Mode: Personal inhalation device Essential oil: Lavender, bergamot, ylang and sweet orange blend (% not specified) Protocol: Ad libitum use during work shifts for 30 days	Placebo inhalation device containing 0.9% saline	STAI	No statistically significant difference in anxiety between intervention and placebo groups.	12/13

Abbreviations: AG, aromatherapy group; CG, control group; PG, placebo group; PSS, perceived stress scale; STAI, state–trait anxiety inventory.

### 2.5 Data Synthesis

A narrative synthesis approach was employed to summarise and interpret the findings of the included studies, given the heterogeneity in study designs, interventions, outcome measures and follow‐up periods. Key characteristics of each study (including sample size, type of aromatherapy, route of administration, duration, frequency and measurement instruments) were tabulated to facilitate comparison. Quantitative outcome data on stress and anxiety were summarised based on reported statistical results, including significance levels, pre–post comparisons and between‐group differences where available. Due to the limited number of studies and methodological variability, meta‐analysis was not conducted. Instead, patterns of effectiveness, consistency of results and methodological quality were considered in interpreting the overall evidence. Potential sources of heterogeneity, including differences in ICU environments, essential oil types and intervention delivery methods, were explored to contextualise the findings.

## 3 Results

### 3.1 Selection of Studies

A total of 279 records were identified through the database search. After removing 118 duplicate records, 161 studies remained for title and abstract screening. Of these, 149 records were excluded because they did not meet the eligibility criteria based on their titles and abstracts. The full texts of the remaining 12 articles were sought for retrieval; however, the full texts of two articles could not be accessed. Consequently, 10 full‐text articles were assessed for eligibility. During the full‐text review, seven studies were excluded for the following reasons: four studies did not evaluate stress or anxiety outcomes, one study did not include ICU nurses as participants, one study did not involve an aromatherapy intervention, and one study had an ineligible study design. In addition, four reports were identified through citation searching and assessed for eligibility; two reports were excluded, and two additional studies met the inclusion criteria. Overall, five studies examining the effects of aromatherapy on stress and anxiety among intensive care unit nurses were included in this systematic review. The study selection process is presented in the PRISMA flow diagram (Figure 1).

### 3.2 Methodological Quality Assessment

Item‐level assessment revealed that both randomised controlled trials demonstrated adequate allocation concealment and reported blinding of outcome assessors, suggesting a low risk of selection and detection bias [20, 21]. Although follow‐up was adequately described in both studies, neither explicitly reported intention‐to‐treat analyses; therefore, this domain was rated as unclear (Table 3). Among the quasi‐experimental studies, Eren and Öztunç [22] and Seo et al. [24] established baseline comparability through the inclusion of comparable participant groups and control conditions. In contrast, Pemberton and Turpin [23] used a self‐controlled pre–post design without an independent control group and showed limitations in outcome measurement reliability and statistical analysis, making it more susceptible to bias (Table 4).

**TABLE 3:** Quality assessment of randomised controlled trials.

	Lee et al. [20]	Hubbard et al. [21]
1. Was true randomisation used for assignment of participants to treatment groups?	Yes	Yes
2. Was allocation to treatment groups concealed?	Yes	Yes
3. Were treatment groups similar at the baseline?	Yes	Yes
4. Were participants blind to treatment assignment?	Yes	Yes
5. Were those delivering the treatment blind to treatment assignment?	Yes	Yes
6. Were treatment groups treated identically other than the intervention of interest?	Yes	Yes
7. Were outcome assessors blind to treatment assignment?	Yes	Yes
8. Were outcomes measured in the same way for treatment groups?	Yes	Yes
9. Were outcomes measured in a reliable way?	Yes	Yes
10. Was follow‐up complete and, if not, were differences between groups in terms of their follow‐up adequately described and analysed?	Yes	Yes
11. Were participants analysed in the groups to which they were randomised?	Unclear	Unclear
12. Was appropriate statistical analysis used?	Yes	Yes
13. Was the trial design appropriate and any deviations from the standard RCT design (individual randomisation, parallel groups) accounted for in the conduct and analysis of the trial?	Yes	Yes
Total score	12	12

*Note:* The quality assessment items are derived from the Joanna Briggs Institute Critical Appraisal Checklist for Randomized Controlled Trials [19].

**TABLE 4:** Quality assessment of quasi‐experimental research.

	Eren and Öztunç [22]	Pemberton and Turpin [23]	Seo et al. [24]
1. Is it clear in the study what is the ‘cause’ and what is the ‘effect’ (i.e., there is no confusion about which variable comes first)?	Yes	Yes	Yes
2. Were the participants included in any comparisons similar?	Yes	Yes	Yes
3. Were the participants included in any comparisons receiving similar treatment/care, other than the exposure or intervention of interest?	Yes	Yes	Yes
4. Was there a control group?	Yes	No	Yes
5. Were there multiple measurements of the outcome both pre and post the intervention/exposure?	Yes	Yes	Yes
6. Was follow‐up complete and if not, were differences between groups in terms of their follow‐up adequately described and analysed?	Yes	Yes	Yes
7. Were the outcomes of participants included in any comparisons measured in the same way?	Yes	Yes	Yes
8. Were outcomes measured in a reliable way?	Yes	No	Yes
9. Was appropriate statistical analysis used?	Yes	No	Yes
Total score	9	6	9

*Note:* The quality assessment items are derived from the Joanna Briggs Institute Critical Appraisal Checklist for Quasi‐Experimental Studies [18].

### 3.3 General Characteristics of the Study and Participants

In the systematic review, five studies with sample sizes ranging from 14 to 120 and a total of 254 participants were deemed suitable for inclusion. It has been reported that in all studies, participants were actively working in an intensive care setting and, for the most part, had at least 1 year of professional experience. The studies included in the systematic review were conducted in three different countries: the United States (*n* = 2), South Korea (*n* = 2) and Turkey (*n* = 1). The included studies were published between 2008 and 2024, with two studies published before 2017 and three studies published from 2017 onwards, reflecting a recent increase in research on aromatherapy among ICU nurses (Table 2).

### 3.4 Measurement Instruments and Assessment Timeline

The included studies utilised a combination of multidimensional scales and visual/numerical analogue scales. Specifically, general anxiety and stress traits were evaluated using the State–Trait Anxiety Inventory (STAI/STAI‐A) in three studies [20, 21, 22] and the Perceived Stress Scale (PSS‐10) in one study [22]. Acute, situation‐specific responses were captured using 10‐cm Visual Analog Scales (VAS) for stress and anxiety [20, 24] or a 1–10 numerical rating scale [22, 23]. Regarding linguistic and cross‐cultural psychometric validity, the instruments were well validated in the respective native languages of the target populations, including Turkish [25, 26] and Korean [27] adaptations. For all instruments (STAI, PSS, VAS‐Stress, VAS‐Anxiety and Numerical Stress Logs), higher scores mathematically indicated greater severity of perceived stress or anxiety, with the exception of the VAS used by Eren & Öztunç [22], which measured the degree of relaxation (where higher scores indicated positive outcomes). The timing of outcome assessments varied substantially, capturing both immediate and cumulative effects. Immediate post‐session effects were evaluated within 10–30 min post‐intervention [22, 24], while shift‐specific effects were monitored before and after a single 2‐h shift [20] or tracked 1 h post‐application across three consecutive 12‐h shifts [23]. Conversely, Hubbard et al. [21] focused on long‐term cumulative outcomes, measuring changes over a prolonged 30‐day intervention window (Table 2).

### 3.5 Study Design and Methodological Characteristics

Upon reviewing the study designs of the included studies, it was determined that two were randomised controlled trials, one was a non‐randomised controlled clinical trial, and two were conducted using a quasi‐experimental design. In randomised controlled trials, participants were assigned to intervention and control groups using simple randomisation methods [20, 21]. The level of blinding varied across studies; one study used a double‐blind design [20], another used a single‐blind design [21], one quasi‐experimental study employed evaluator blinding [24], while other quasi‐experimental studies did not use blinding (Table 2).

### 3.6 Characteristics of Aromatherapy Intervention

The included studies employed diverse aromatherapy interventions, with variations in essential oil type, concentration, administration route, duration and frequency. In three studies, inhalation was the primary mode of delivery [20, 21, 22], while the remaining two studies used topical application and gargle methods [23, 24]. *Lavandula angustifolia* (lavender) was used in three studies [21, 22, 23], reflecting its popularity in stress and anxiety management. Other essential oils included *Origanum majorana* L. (marjoram) [20], *Salvia sclarea* (clary sage) [23], *Citrus bergamia* (bergamot), *Cananga odorata* (ylang ylang), *Citrus sinensis* (sweet orange) [21] and a combination of peppermint, lemon, tea tree and ylang ylang in a gargle solution [24]. Intervention duration varied from single‐session exposures [20, 22] to multiple sessions across several work shifts [23] and daily repeated applications for three consecutive days [24]. In one study, aromatherapy was administered throughout working hours using a personal inhalation device, allowing participants to self‐administer the intervention as frequently as desired. For inhalation‐based interventions, essential oils were either placed on cloths near participants' breathing zones [20, 22] or delivered via personal inhalation devices with voluntary use throughout work hours [21]. Topical applications were performed on the inner forearm using carrier oils [23], whereas the gargle intervention involved daily oral rinses for 15–20 s [24] (Table 2).

### 3.7 Comparison Group

Across the included studies, different types of control conditions were employed. In two studies, almond oil was used as an active control condition, with the same application protocol, duration and setting as the intervention groups [20, 23]. In one study, sunflower oil was used as an active control under identical physical and environmental conditions [22]. One study utilised a placebo control involving 0.9% sodium chloride administered via an inhalation device, matching the intervention conditions in duration and procedure [21]. Another study included two control groups: a placebo group performing gargling with normal saline under the same conditions as the intervention group, and a no‐intervention control group, allowing comparison with both placebo and usual care conditions [24] (Table 2).

### 3.8 The Effect of Aromatherapy on Stress and Anxiety

Stress was evaluated in four studies, with three reporting significant reductions following aromatherapy interventions [20, 23, 24], while one study found no significant effect on perceived stress [22]. The interventions associated with reduced stress included inhalation of *Origanum majorana* essential oil, topical application of lavender and clary sage oils and an aromatherapy gargle containing peppermint, lemon, tea tree and ylang–ylang essential oils.

Anxiety was assessed in three studies [20, 21, 22]. Two studies reported reductions in anxiety following aromatherapy interventions [20, 22]. Specifically, Lee et al. [20] found significant decreases in anxiety after inhalation of *Origanum majorana* essential oil. Eren and Öztunç [22] reported significant reductions in state anxiety following both lavender and sunflower oil inhalation; however, no significant effects were observed for trait anxiety, and the reductions in state anxiety were not specific to the aromatherapy intervention. In contrast, Hubbard et al. [21] found no statistically significant differences in anxiety between participants receiving an essential oil blend and those receiving placebo inhalation (Table 2).

### 3.9 Adverse and Undesired Effects

Adverse or undesired effects were assessed in four of the five studies included in this systematic review [21, 22, 23, 24]. Safety monitoring procedures varied considerably across studies. Eren and Öztunç [22] predefined study withdrawal criteria, including allergic reactions, respiratory symptoms, coughing and nausea following inhalation. Although no serious adverse events or study discontinuations were reported, some participants experienced minor adverse effects, including headache (*n* = 2), nausea (*n* = 1) and eye irritation (*n* = 1). In addition, two participants reported that the aroma of the essential oil was excessively strong, despite describing a subjective sense of relaxation following the intervention. Pemberton and Turpin [23] conducted pre‐intervention skin patch testing to identify potential irritation or sensitivity to the essential oils used. Hubbard et al. [21] implemented structured safety monitoring throughout the 30‐day intervention period by administering safety surveys on days 7, 14 and 21 to assess adverse reactions and intervention‐related discontinuation. Seo et al. [24] excluded participants with known allergies to essential oils; however, detailed adverse‐event monitoring procedures were not reported. Across the included studies, no serious adverse events, severe allergic reactions or participant withdrawals attributable to aromatherapy were reported. Nevertheless, the reporting of safety outcomes was generally limited and inconsistent, and several studies did not clearly specify whether adverse events were actively monitored or passively reported.

## 4 Discussion

To the best of our knowledge, this study is the first systematic review to examine the effects of aromatherapy on stress and anxiety among nurses working in intensive care units. One of the most relevant studies on this topic in the literature is the systematic review and meta‐analysis by Xu et al. [14], which included all hospital nurses. The study in question demonstrated that aromatherapy significantly reduced stress among nurses; however, it was reported that evidence regarding its effects on anxiety was insufficient and that the results were inconsistent. However, in the study by Xu and colleagues, nurses were treated as a heterogeneous sample drawn from various clinical units, and the working conditions specific to the intensive care unit were not evaluated as a separate subgroup. However, intensive care nurses are exposed to unique stressors such as constant alarm sounds, excessive workloads, monitoring critically ill patients and frequent encounters with patient deaths; these conditions can have a more pronounced effect on anxiety and the stress response [1, 28, 29]. In this context, the present review, which includes only intensive care nurses, makes a significant contribution to the literature by evaluating the effects of aromatherapy in a more homogeneous and clinically high‐risk group. The findings from the present review suggest that aromatherapy may have potential as a complementary intervention for intensive care nurses; however, conclusions should be interpreted cautiously given the limited number of available studies.

Previous reviews have reported beneficial effects of aromatherapy on stress and anxiety among nurses and healthcare professionals in diverse clinical settings [12, 13, 30, 31]. In the present review, evidence for stress reduction was somewhat more consistent, whereas evidence for anxiety reduction was mixed and limited by small sample sizes, heterogeneous interventions and inconsistent between‐group effects. Accordingly, any potential effect of aromatherapy on anxiety should be interpreted cautiously until confirmed by larger, well‐designed randomised controlled trials using standardised intervention protocols and outcome measures. The potential stress‐reducing effects of aromatherapy have been attributed to several physiological and psychological mechanisms. According to the narrative compilation by Men et al. [32], essential oil molecules enter the bloodstream via the olfactory pathway and transdermal absorption, thereby regulating the nervous, circulatory and immune systems. In particular, modulation of the limbic system and enhanced GABAergic neurotransmission have been proposed as mechanisms that may contribute to anxiolytic effects. Given the stressors in an intensive care setting, such as constant alarm, high cognitive load and the responsibility of managing critically ill patients, aromatherapy may represent a complementary intervention with potential to alleviate occupational stress. However, despite the reported therapeutic benefits of aromatherapy, caution should be exercised when using it. Essential oils can cause unwanted effects such as skin irritation, allergic reactions and, in some cases, interactions with medications. For this reason, ensuring appropriate dilution ratios, using proper application methods and consulting with healthcare professionals are of great importance for both ensuring safety and enhancing efficacy [33]. Although no serious adverse events or intervention‐related withdrawals were reported in the included studies, the available evidence regarding safety should be interpreted cautiously because adverse‐event monitoring procedures were inconsistent across studies and the sample sizes were generally small.

When examining the intervention characteristics across the included studies, substantial heterogeneity was observed in essential oil type, delivery route, duration, frequency of administration, outcome measures and study design. Although inhalation was the most commonly used delivery method and may be particularly feasible in intensive care settings due to its simplicity, rapid administration and minimal disruption to workflow, the current evidence is insufficient to determine an optimal aromatherapy protocol for ICU nurses. Lavender was the most frequently investigated essential oil and has been associated with stress reduction in previous systematic reviews and meta‐analyses [34, 35]. The potential anxiolytic and stress‐reducing effects of lavender may be explained by its action on the central nervous system, particularly through modulation of the limbic system, which plays a key role in emotional regulation. In addition, linalool and linalyl acetate, key constituents of lavender oil, have been suggested to potentially enhance inhibitory neurotransmission, including GABAergic activity, thereby contributing to reduced sympathetic activation and improved relaxation responses [36]. However, other oils such as marjoram, bergamot, ylang ylang, sweet orange, clary sage, peppermint, lemon and tea tree were also used across studies, making direct comparisons difficult. Similarly, interventions ranged from single‐session administration to repeated applications across several shifts or extended periods, and outcome assessment relied on heterogeneous measurement tools, including VAS, numerical rating scales, PSS and STAI variants. Consequently, while inhalation‐based aromatherapy appears to be a potentially feasible and promising approach in ICU environments, current evidence does not allow conclusions regarding the most effective oil type, dosage, duration, frequency or delivery method. These uncertainties highlight the need for standardised intervention protocols and methodologically rigorous future trials.

Although most included studies met the majority of the JBI appraisal criteria, methodological quality should not be interpreted solely on the basis of numerical checklist scores. Certain domains, such as randomisation procedures, allocation concealment, presence of an independent control group, blinding and appropriate analysis of randomised participants, have greater implications for internal validity than others. In the present review, the randomised controlled trials generally provided stronger evidence than the quasi‐experimental and self‐controlled studies. Conversely, findings from studies without independent control groups or with greater susceptibility to bias should be interpreted as supportive and exploratory rather than confirmatory. Consequently, although the overall findings suggest potential benefits of aromatherapy for stress reduction among ICU nurses, confidence in the evidence remains moderate because of methodological limitations and the predominance of non‐randomised study designs.

## 5 Limitations

This systematic review limits the generalisability of the findings due to small sample sizes and the limited number of studies specific to intensive care nurses. Differences in the types of essential oils, application methods, duration and frequency of treatment and measurement tools make it difficult to directly compare studies. In some studies, inadequate blinding and short follow‐up periods hinder the assessment of the long‐term effects of aromatherapy. Furthermore, the fact that the study was conducted in only a limited number of countries and included only English‐language publications reduces the generalisability of the findings to different cultural and clinical contexts. Furthermore, although four major databases were searched, other databases with strong coverage of nursing, psychological and allied health literature (e.g., CINAHL, Embase, PsycINFO and ProQuest) were not included. Consequently, it is possible that some relevant studies may have been missed despite the comprehensive search strategy and manual citation searching.

## 6 Recommendations or Implications for Practice and Further Research

This systematic review suggests that aromatherapy may represent a feasible complementary approach for supporting the well‐being of ICU nurses; however, routine implementation should await stronger evidence from high‐quality randomised controlled trials. Aromatherapy may be considered as an optional supportive component within institutional programs aimed at promoting nurses' occupational health and well‐being in intensive care units, provided that individual preferences, scent sensitivity and infection‐control considerations are taken into account. Ethical considerations should also be addressed when implementing workplace‐based aromatherapy interventions. Participation should be voluntary and based on informed consent, with confidentiality protected throughout the process. Staff should have the right to decline participation without consequences, and care should be taken to avoid perceived pressure in hierarchical workplace settings. In addition, individual factors such as fragrance sensitivity, allergies, asthma, pregnancy and other relevant health conditions should be considered before implementing aromatherapy interventions. However, recommendations regarding safety should be interpreted cautiously because adverse‐event monitoring was inconsistently reported across studies and the available evidence is based on relatively small samples. In future studies, randomised controlled trials with sufficient sample sizes and high methodological quality should be conducted to more clearly demonstrate the effects of aromatherapy; intervention protocols (type of oil, dose, duration and application method) should be standardised, and long‐term effects should be evaluated across different shift schedules. In addition, qualitative studies can support the quantitative findings by revealing intensive care nurses' experiences and perceptions regarding aromatherapy.

## 7 Conclusion

This systematic review suggests that aromatherapy may have potential benefits for reducing stress among intensive care unit nurses; however, evidence regarding anxiety remains mixed. Overall, the available evidence should be interpreted with caution because of the small number of included studies, limited sample sizes, variability in study designs and methodological quality and substantial heterogeneity in intervention protocols, essential oil types and outcome measures. Consequently, the current evidence is insufficient to recommend routine clinical implementation or to identify an optimal aromatherapy protocol. Future well‐designed randomised controlled trials with standardised intervention protocols, consistent outcome measures and longer follow‐up periods are needed to strengthen the evidence base.

## Funding

The authors have nothing to report.

## Ethics Statement

As this is a review of published literature, no ethics approval was required, and patient consent lies with the original publication.

## Conflicts of Interest

The authors declare no conflicts of interest.

## Supporting information


**File S1:** Complete database‐specific search strategies.

## Data Availability

The data that support the findings of this study are available on request from the corresponding author. The data are not publicly available due to privacy or ethical restrictions.
