# Supplementary material for: Effects of Aromatherapy on Stress and Anxiety Among Intensive Care Unit Nurses: A Systematic Review
Source: Nurs Crit Care. 2026 Sep 24;31(6):e70705. doi: 10.1111/nicc.70705 (PMC13613012; doi:10.1111/nicc.70705)
Supplement: Supplementary file 1 [file NICC-31-0-s001.docx]

**Supplementary File 1. Complete database-specific search strategies**

**PubMed**

(("intensive care unit*" OR ICU OR "critical care")

AND

(nurse* OR nursing)

AND

(aromatherapy OR "essential oil*" OR lavender))

**Scopus**

(("intensive care unit*" OR ICU OR "critical care")

AND

(nurse* OR nursing)

AND

(aromatherapy OR "essential oil*" OR lavender))

**Web of Science**

(("intensive care unit*" OR ICU OR "critical care")

AND

(nurse* OR nursing)

AND

(aromatherapy OR "essential oil*" OR lavender))

**Cochrane Library**

(("intensive care unit*" OR ICU OR "critical care")

AND

(nurse* OR nursing)

AND

(aromatherapy OR "essential oil*" OR lavender))
